# Supplementary material for: Antioxidative and Antiglycative Stress Activities of Selenoglutathione Diselenide
Source: Pharmaceuticals (Basel). 2024 Aug 9;17(8):1049. doi: 10.3390/ph17081049 (PMC11359168; doi:10.3390/ph17081049)
Supplement: Supplementary file 1 [file pharmaceuticals-17-01049-s001.zip › pharmaceuticals-3098931-supplementary.pdf]

Supporting Materials

Antioxidative and antiglycative stress activities of selenoglutathione diselenide

Akiko Kanamori*, Nana Egawa, Suyako Yamasaki, Takehito Ikeda, Marcia Juciele da Rocha, Cristiani Folharini Bortolatto, Lucielli Savegnago, César Augusto Brüning and Michio Iwaoka*

1. Preparation of GSeSeG

GSeSeG was prepared by liquid-phase peptide synthesis (LPPS) according to our previous protocol (Scheme S1) [17]. Briefly, H-Gly-O*t*Bu was reacted with Fmoc-Sec(Se-PMB)-OH (**1**) using EDCI and HOBt as coupling reagents in DMF. The obtained Fmoc-Sec(Se-PMB)-Gly-O*t*Bu (**2**) was then treated with Et_2NH in 20 % DCM/DMF to deprotect the Fmoc group. The resulting dipeptide was coupled with Boc-Glu-O*t*Bu using EDCI and HOBt in DMF to yield **3**. The obtained tripeptide **3** was treated with I_2 and then aqueous TFA to afford GSeSeG in total 90 % yield. The purity of GSeSeG was confirmed by HPLC analysis (see below).

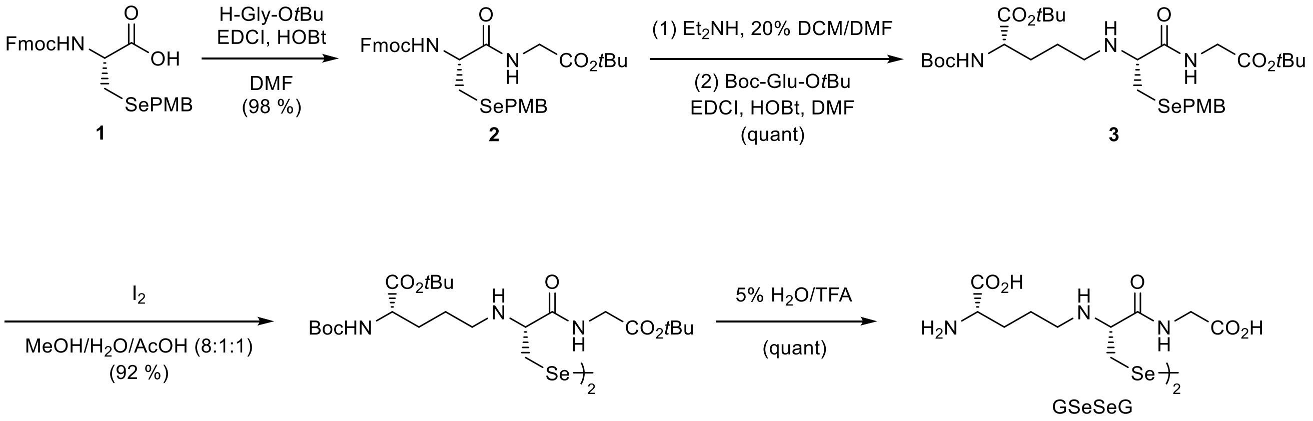

Scheme S1. Synthesis of GSeSeG [17].

2. RP-HPLC analysis of GSeSeG

The purity of GSeSeG was analyzed by RP-HPLC using a Tosoh ODS-100V (4.6 mm x 150 mm) column, which was equilibrated with 0.1 % TFA in H_2O (solvent A) at 35 °C at a flow rate of 0.7 mL/min. The sample solution was prepared by dissolving GSeSeG (1 mM) in a phosphate-buffered saline at pH 7.4 (PBS). After the sample injection, a solvent gradient was applied by increasing the ratio of solvent B (0.1 % TFA in acetonitrile) from 0 % to 12 % in 31 min and then 12 % to 14 % in 10 min. The ratio was kept constant after the retention time of 41 min. GSeSeG was detected at the wavelength of 210 nm.

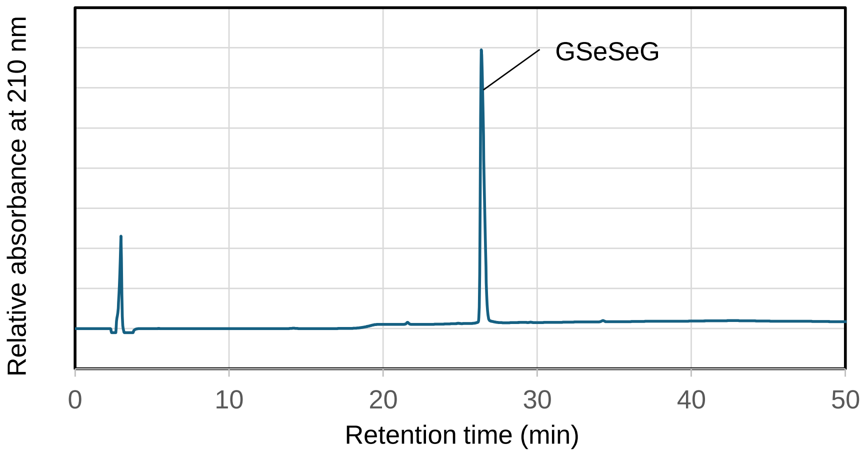

Figure S1. The RP-HPLC chromatogram for GSeSeG.

3. Preparation of GSeH from GSeSeG

GSeH was generated from GSeSeG (0.5 mM) by reacting with NADPH (1.5 mM) and GR (10 unit/mL) in PBS at 37 °C in 10 min. The HPLC analysis conditions were the same as Figure S1.

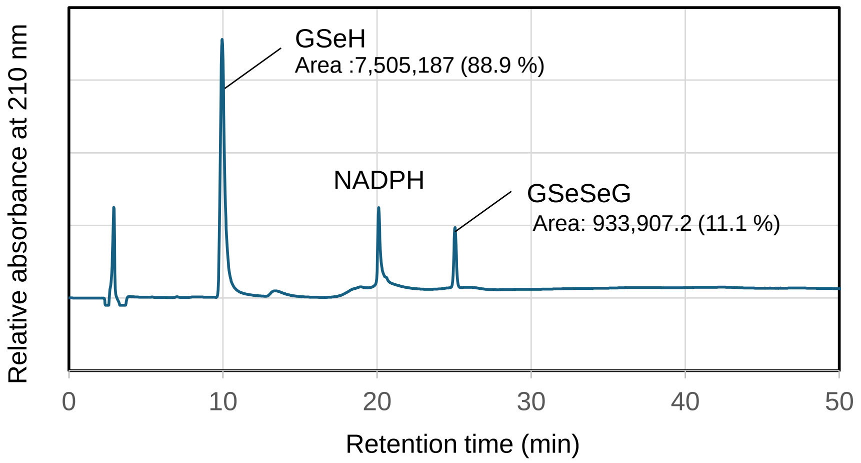

Figure S2. The RP-HPLC chromatogram for GSeH.

4. RP-HPLC charts for the assay solutions of GLO 1-like MG degradation activities of GSeH.

Raw data for the chromatograms of Figure 2B are shown below.

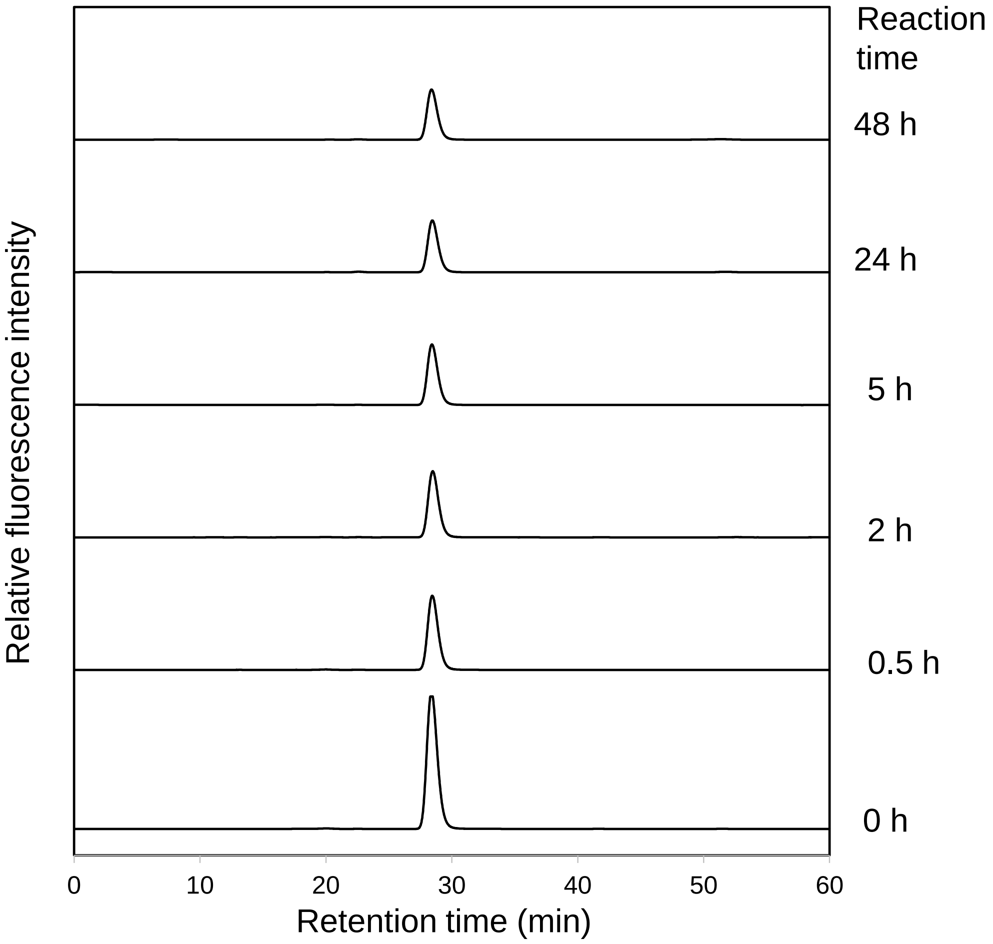

Figure S3. The RP-HPLC chromatograms for the MG-MDB adduct generated by the reaction of GSeH (1 mM) and MG (0.5 mM) with a different reaction time.

Reference

[17] Shimodaira, S.; Asano, Y.; Arai, K.; Iwaoka, M. *Biochemistry* **2017**, *56*, 5644–5653.
